# Astrocyte Apoptosis and HIV Replication Are Modulated in Host Cells Coinfected with *Trypanosoma cruzi*

**DOI:** 10.3389/fcimb.2017.00345

**Published:** 2017-08-02

**Authors:** Javier M. Urquiza, Juan M. Burgos, Diego S. Ojeda, Carla A. Pascuale, M. Susana Leguizamón, Jorge F. Quarleri

**Affiliations:** ^1^Consejo Nacional de Investigaciones Científicas y Técnicas Buenos Aires, Argentina; ^2^Instituto de Investigaciones Biomédicas en Retrovirus y Sida, Universidad de Buenos Aires, Consejo Nacional de Investigaciones Científicas y Técnicas Buenos Aires, Argentina; ^3^Instituto de Investigaciones Biotecnológicas, Universidad Nacional de San Martín, San Martín, Argentina Consejo Nacional de Investigaciones Científicas y Técnicas Buenos Aires, Argentina

**Keywords:** *Trypanosoma cruzi*, HIV, coinfection, astrocytes, apoptosis, Chagasic meningoencephalitis

## Abstract

The protozoan *Trypanosoma cruzi* is the etiological agent of Chagas disease. In immunosuppressed individuals, as it occurs in the coinfection with human immunodeficiency virus (HIV), the central nervous system may be affected. In this regard, reactivation of Chagas disease is severe and often lethal, and it accounts for meningoencephalitis. Astrocytes play a crucial role in the environment maintenance of healthy neurons; however, they can host HIV and *T. cruzi*. In this report, human astrocytes were infected *in vitro* with both genetically modified-pathogens to express alternative fluorophore. As evidenced by fluorescence microscopy and flow cytometry, HIV and *T. cruzi* coexist in the same astrocyte, likely favoring reciprocal interactions. In this context, lower rates of cell death were observed in both *T. cruzi* monoinfected-astrocytes and HIV-*T. cruzi* coinfection in comparison with those infected only with HIV. The level of HIV replication is significantly diminished under *T. cruzi* coinfection, but without affecting the infectivity of the HIV progeny. This interference with viral replication appears to be related to the *T. cruzi* multiplication rate or its increased intracellular presence but does not require their intracellular cohabitation or infected cell-to-cell contact. Among several Th1/Th2/Th17 profile-related cytokines, only IL-6 was overexpressed in HIV-*T. cruzi* coinfection exhibiting its cytoprotective role. This study demonstrates that *T. cruzi* and HIV are able to coinfect astrocytes thus altering viral replication and apoptosis.

## Introduction

Chagas disease is a chronic, infectious entity caused by the protozoan *Trypanosoma cruzi* (*T. cruzi*). This disease is highly prevalent in Latin America and increasingly widespread in developed countries (WHO, 2017a). According to the World Health Organization (WHO) an estimated 8 million people are infected worldwide, and 25 million people are at-risk of acquiring *T. cruzi* infection (WHO, 2017b).

*Trypanosoma cruzi* parasites are classified into six groups (Discrete Typing Units, DTU I to VI) based on genomic and molecular markers (Zingales et al., 2009; Cura et al., 2012). These groups share a phylogenetic relationship with some eco-epidemiological, biological, and clinical defined behaviors (Zingales et al., 2012). The parasite life cycle comprises the infective bloodstream form (trypomastigote) able to infect different nucleated cells. This complex process includes the parasite-cell contact, an endocytic process with alteration of cellular cytoskeleton, and development of a large vacuole (parasitophorous vacuole) that hosts trypomastigotes. After escape from it, the transformation to the amastigote form occurs which multiply by binary division into the cytoplasm. During this stage, the parasite maintains metabolic dependence on nucleotide and fatty acid/glucose metabolisms, cellular energy, and Akt-mediated prosurvival signaling (Caradonna et al., 2013). At last, amastigotes differentiate into trypomastigotes, host cell are lysed, and infective parasites are release to the bloodstream. The acute phase of Chagas disease is characterized by high parasitemia, broad tissue parasitism, and the development of different evasion mechanisms that impair the specific signoificnalty diminished immune response (Dosreis, 2011). Although, the host immune system fails to eliminate the parasite, it controls parasitemia when the chronic asymptomatic phase is achieved (Coura and Borges-Pereira, 2010; Borges et al., 2016).

During the evolution of the infection in immunocompetent patients, around 30% develop myocarditis, megaesophagus, and/or megacolon as the main manifestations of Chagas disease (Kirchhoff et al., 2004). The involvement of the central nervous system (CNS) is a severe life-threatening condition infrequent in immunocompetent patients. However, this manifestation can arise, as Chagasic meningoencephalitis, during the chronic phase of infection in immunocompromised hosts, usually as a disease reactivation (Bern et al., 2011). On immune-suppression therapy, and in the context of human immunodeficiency virus/acquired immune deficiency syndrome (HIV/AIDS) Chagas disease reactivation lead to a severe and often lethal outcome (Pinazo et al., 2013). Interactions between parasitic infections and HIV/AIDS have been reported as well as the detrimental impact on their natural history (Da Costa, 2000; Harms and Feldmeier, 2002; Sartori et al., 2002). Chagasic meningoencephalitis is characterized by brain nodular reaction involving neutrophils, microglia, astrocytes, and perivascular lymphocytic infiltrate in various foci along the CNS (Lattes and Lasala, 2014). Astrocytes, most abundant cells in brain that maintain an adequate environment for neurons, have multiple functions including endocytosis and antigen presentation (Jensen et al., 2013). As other cells from nervous system, astrocytes can host several infectious agents, including HIV and *T. cruzi* (Blanchet et al., 2010; Vargas-Zambrano et al., 2013) pointing them as important performers in Chagasic meningoencephalitis development.

According to our knowledge, at present, there are no *in vitro* studies describing interactions between *T. cruzi* and HIV in CNS cells. Since both pathogens are able to infect and replicate within astrocytes, we demonstrate the cellular coexistence of *T. cruzi* and HIV as well as the impact on both HIV replication efficiency and cell death.

## Materials and methods

### Cell lines and *Trypanosoma cruzi* culture

A human astrocytoma cell line (U373 MAGI; NIH-AIDS Reagents Program Cat# 3595) was used and maintained in Dulbecco's modified Eagle medium (DMEM) supplemented with 10% fetal bovine serum (FBS), 50 U of penicillin/ml, and 50 mg of streptomycin/ml (Sigma-Aldrich). This cell line was obtained from the AIDS Reagent Program, National Institutes of Health (NIH), USA. Three *T. cruzi* parasites were used: CL (DTU VI), K98 (DTU I), and Sylvio (Sy, DTU I). The K98 strain was also genetically modified to express the enhanced green fluorescent protein (eGFP). Parasites were maintained in monolayers of Vero cells. Trypomastigotes were harvested from supernatants by low speed centrifugation (500 rpm, 10 min) after being released from infected cells aimed at removing any contaminating cell debris. Then, parasites were pelleted (2,500 rpm 10 min) and finally resuspended in DMEM supplemented with 10% FBS, 50 U of penicillin/ml, and 50 mg of streptomycin/ml (Andreani et al., 2009; Aridgides et al., 2013). Parasites were counted in a Neubauer chamber.

### Preparation and titration of HIV-1 env-pseudotyped viruses, and reporter virus generation

pNL4-3 is a full-length infectious molecular clone of HIV-1 exhibiting X4 tropism. The NL4-3-GFP molecular clone contains the enhanced version of the green fluorescent protein gene (eGFP) and an internal ribosome entry-site (IRES) inserted upstream from the functional nef reading frames in the NL4-3 clone. An IRES element between the reporter gene and the HIV-Nef drives the expression of eGFP. For this study, the additional pNL4-3-DsRed (*Discosoma* sp. red fluorescent protein) reporter virus was constructed by substituting the region of pNL4-3-GFP which contains the eGFP gene instead of the DsRed gene from Clontech. High-titer stocks of HIV-1-pseudotyped with vesicular stomatitis virus envelope glycoprotein (VSV-G) was constructed by cotransfecting 293T cells using a VSV-G expression plasmid (Clontech) at HIV/VSV-G plasmid ratio of 10:1. The supernatant was harvested 48 and 72 h, filtered through a 0.45 μm filter and concentrated for over 5 h at 18,000 rpm. The pellet was resuspended in DMEM supplemented with 10% FBS and stored at −86°C until use. The titer of virus stocks was adjusted to 100 ng/μl of p24 (HIV capsid protein). To determine whether HIV replication correlated with GFP or DsRed expression in astrocytes, a time-course analysis was performed following *in vitro* infection of astrocytes with either HIV-GFP or HIV-DsRED. Three different variables were monitored as a function of time: (1) p24 concentration in cell culture supernatants (p24 ELISA kit, INNOTEST® HIV Antigen mAb), (2) p24 intracellular expression using the KC57 monoclonal antibody against the HIV p24 capsid protein (see below), and (3) HIV gene expression by GFP and DsRED measurement by flow cytometry (FACSCanto II flow cytometer, BD Biosciences, San Jose, CA, USA). Besides, the flow cytometry analysis of cell fluorescence, as a reflection of GFP or DsRED expression, allows the identification of productively infected cells for each pathogen, eventual cohabitation of both, and neighbor-uninfected cells in a heterogeneous population. The flow cytometer sensitivity and performance was checked prior to data acquisition using Cytometer Setup & Tracking beads (BD Biosciences, San Jose, CA, USA).

### Timeline of HIV/*Trypanosoma cruzi* co-infection assays

Human astrocytes were seeded in 48-well plates (3 × 10^4^/well) or Nunc® Lab-Tek II chamber slides (10^3^/well). Then, they were exposed separately to *T. cruzi* and HIV-1 at an interval of 24 h, according to two alternative schemes: (i), *T. cruzi* before HIV (Tc/HIV), and (ii) HIV before *T. cruzi* (HIV/Tc). For both schemes, prior to second pathogen exposure, astrocytes were washed five times with fresh DMEM media to remove free pathogens. Two HIV inocula were used (8, or 0.8 μg/ml measured as p24 antigen), while the infection with *T. cruzi* trypomastigotes was carried out in a cell:parasite ratio of 1:5. Under these conditions, *T. cruzi* infected-astrocytes appeared to release trypomastigotes since 5 days post-infection (dpi). After *T. cruzi* exposure, all measurements were performed in cells and culture supernatants at 3 and 5 dpi in triplicate.

All experiments were carried out in a BSL-3 laboratory at the INBIRS. Following institutional rules, all biological materials are mandatorily inactivated by autoclaving prior to disposal by incineration. Incineration is carried out in a high-temperature incinerator.

### *In vitro* HIV and *T. cruzi* infection: analysis by flow cytometry

The detection of intracellular HIV-1 p24 antigen was performed using a monoclonal antibody (mAb) to the HIV-1 core antigen labeled with fluorescein isothiocyanate (p24-FITC), following the manufacturer's recommended amount for all intracellular staining. This mAb (KC57; Beckman Coulter, USA) identifies the 55, 39, 33, and 24 kDa species of the core antigens of HIV-1 (Chassagne et al., 1986; Darden et al., 2000).

Following infection with the DsRED or GFP-expressing virus, and GFP-expressing parasite, the specific fluorescence was measured. Cells were gated on the basis of side scatter and forward scatter for debris exclusion. Subsequently, infected cells were identified by their green or red fluorescence and evaluated by different apoptosis assays (see below). Data from 5 × 10^4^ cells were collected, stored, and analyzed with FlowJo X software for Windows 7.0.

### Astrocyte apoptosis measurement

Two different flow cytometry assays were performed in order to evaluate astrocyte apoptosis.

#### Annexin-V/7AAD staining

Plasma membrane permeability and phosphatidylserine cell translocation were measured by flow cytometry in order to define the percentage of early apoptotic cells. To this aim, dual staining was performed with PE or APC-conjugated Annexin-V and 7-amino-actinomycin D (7AAD) using the Annexin-V/7AAD apoptosis detection kit (BD Biosciences). Early apoptotic cells were defined as Annexin-V+/7AAD− cells while those Annexin-V+/7AAD+ represented necrotic cells. Percentages of Annexin-V and 7AAD were evaluated 3 and 5 dpi.

#### Caspase 3/7 detection

The Vybrant® FAM Caspase-3 and -7 Assay Kit uses an approach to detect active caspases based on a fluorescent inhibitor of caspases (FLICA™), essentially an affinity label. The provider's protocols were followed. For simultaneous evaluation of caspases activation and membrane permeability, dual staining with the FLICA reagent specific for caspase-3 and -7 and 7AAD were evaluated by flow cytometry using a FACSCanto II (BD Biosciences). We evaluated the activities of caspase-3 and -7 at 3 and 5 dpi.

### Assessment of the infectivity of the HIV progeny using GHOST cells

The infectivity of the HIV progeny was measured in supernatants by the single-cycle infectivity assay in GHOST-CXCR4 cells (Vodros and Fenyo, 2005). These are human osteosarcoma cells that express a reporter gene (GFP) under the control of the long terminal repeat (LTR) promoter of HIV-2. The GFP expression occurs by Tat transactivation during HIV-1 infection. These cells were maintained in DMEM supplemented with 10% FBS, G418 (500 μg/ml), hygromycin (100 μg/ml), and puromycin (1 μg/ml). Firstly, the p24 level in supernatants was measured from HIV cultured in the presence and absence of *T. cruzi*. Then, the p24 concentration was adjusted to 100 ng/ml. Finally, the viral infectivity was evaluated using GHOST cells from the supernatants. The proportion of GFP-positive cells was measured by flow cytometry.

### Quantification of cytokines in astrocyte culture supernatants

Cytokines IL-2, IL-10, IL-4, IL-6, IFN-γ, TNF-α, and IL-17A were simultaneously measured 4 dpi with *T. cruzi* by flow cytometry (BD FACSCanto flow cytometer, Biosciences, San Jose, CA, USA) in culture supernatants using a cytometric bead array assay (CBA) (Moncunill et al., 2013). The human Th1/Th2/Th17 kit (BD Biosciences, San Jose, CA, USA) was applied following manufacturer's instructions. As reported previously, the limits of detection of each cytokine are 0.1 pg/ml (IL-2), 0.03 pg/ml (IL-4), 1.4 pg/ml (IL-6), 0.5 pg/ml (IFN-γ), 0.9 pg/ml (TNF-α), 0.8 pg/ml (IL-17A), and 16.8 pg/ml (IL-10) (Souto et al., 2014). Quantitative analyses were performed using FCAP Array v1.0.1 software (Soft Flow Inc., Pecs, Hungary). Four days after *T. cruzi* infection, the level of IL-1β in supernatants was quantified for each condition (BD cytokines ELISA Kit).

IL-6 activity was blocked using a neutralizing monoclonal antibody (mouse anti-human IL-6, R&D Systems Inc. Minneapolis, MN) which was added to the culture at 0.2 μg/ml 1 day after HIV exposure. Cells were also incubated with isotype control immunoglobulin G1 (R&D Systems Inc. Minneapolis, MN).

### Astrocytes exposure to recombinant *Trypanosoma cruzi trans*-sialidase (TS) protein (enzymatically active, and inactive isoforms)

TS was expressed in *Escherichia coli* BL21 and purified to homogeneity by immobilized metal affinity chromatography through Ni^2^-charged Hi-Trap chelating columns (GE Healthcare) and ion-exchange chromatography (Mono Q; GE Healthcare) as described previously, followed by passage through a polymyxin column (Pierce) for endotoxin depletion (Leguizamon et al., 1999). As described above (Section Timeline of HIV/*Trypanosoma cruzi* Co-infection Assays), astrocytes were exposed to 1 μg/ml of TS (active, or inactive isoform) according the Tc/HIV timeline (**Figure 2A**) replacing trypomastigotes.

### *In vitro* herpes *Simplex*-2 virus (HSV-2) and *T. cruzi* infection: viral replication measurement

#### Analysis by plaque-forming-units assay in vero cells

Vero cells (African green monkey kidney cells) were cultured in DMEM 5% SFB at a concentration of 3 × 10^6^ per well. Cells infection with *T. cruzi* trypomastigotes was carried out in a cell:parasite ratio of 1:5 when 80% confluence was achieved. Vero cells were washed five times with fresh DMEM media to remove free parasites and 24 h after *T. cruzi* infection were exposed to HSV-2 (G-strain) using different multiplicity of infection −m.o.i- in 300 μl of DMEM. After 2 h cells were washed with PBS and cultured in DMEM 10% plus 0.8% methylcellulose restrictive media. After 48 h, cells were fixed with 1% paraformaldehyde for an hour and then stained with crystal violet. Cytophatic effect (cell lysis) was semi-quantitatively evaluated (from no visible [0] to maximum [++++]). This assay was carried out by duplicate.

#### HSV-2 DNA quantification by qPCR

Total DNA was obtained from 200 μl supernatants of 1 × 10^6^ Vero cells infected with 1 × 10^4^ plaque forming units (pfu) of HSV-2 using PureLink DNA viral extraction kit (Thermo Scientific). Quantitative real-time PCR was performed using SYBR Green PCR Master Mix (Invitrogen) in 20 μl reaction. All primers were used at a concentration of 300 nM. Primers for HSV-2 were 5′-CGCATCATCTACGGGGACACGGA-3′ (forward) and 5′-ATGACGCCGATGTACTTTTTCTT-3′ (reverse). Reactions were carried out in StepONE Plus cycler (Thermo Scientific). The cycling program used was 95°C for 10 min followed by 40 cycles of 95°C for 15 s and 60°C for 60 s. Threshold values (Ct) were used to compare directly. Values are expressed as fold-change compared to the control group.

### Assay to evaluate benznidazole effect on apoptosis and viral replication in astrocytes exposed to *T. cruzi* and HIV

Astrocytes cultured at 37°C and infected at a cell:parasite ratio of 1:5 were incubated following identical conditions as described previously, and then washed five times with fresh DMEM media. Infected astrocytes were cultured and treated for 24 h with 10 μM of benznidazole (Laboratorio ELEA, Argentina), and washed again with fresh DMEM media. Then, these cells were exposed to HIV following the timeline described previously.

### Assessment of *T. cruzi* effects on HIV-1 replication using transwell assays

The ability of *T. cruzi* infected-astrocytes and/or *T. cruzi* free-trypomastigotes to inhibit HIV-1 replication through soluble mechanisms was evaluated using a transwell strategy. To achieve this goal, culture plate inserts were used with membrane-based devices for use with standard plastic cell culture plates. The inserts are made of a polycarbonate membrane in polystyrene plastic holders with 0.4 μm diameter pore μm which allows different conditions for coexistence without intercellular contact. The infection strategy was as follows: HIV-infected astrocytes at the bottom chamber, and the inserts containing astrocytes infected with *T. cruzi, T. cruzi* free-trypomastigotes. In both chambers, uninfected cells were used as controls.

### Fluorescence microscopy

Astrocytes were cultured in Nunc® chamber slides and infected alternatively, by two *Trypanosoma cruzi* parasites (K98 or, CL) in the presence or absence of HIV. Cells were washed thrice to remove free parasites, and then labeled dually for HIV (p24 KC57-FITC antibody, Beckman Coulter) and *T. cruzi* at 2, 4, 6, and 8 dpi. The presence of intracellular amastigotes was evaluated using rabbit polyclonal anti-*T. cruzi* antibody (kindly donated by Dr. Federico Penas, Instituto de Microbiología y Parasitología Médica -IMPAM- Facultad de Medicina, Universidad de Buenos Aires, Argentina). Then, a commercial goat anti-rabbit IgG (H+L) secondary antibody, Alexa Fluor 647 (Invitrogen, Thermo Fisher) was used. Nuclei were stained with DAPI. The coverslips mounted with DAPI Fluoromount-G (SouthernBiotech) were studied in a Nikon Eclipse Ti-S L100 fluorescence microscope using a Plan Apochromat 60 × 1.42 NA oil immersion objective. Images were analyzed using the NIS-Element software.

### Statistical analysis

Where applicable, the two-sided Student's *t*-test was used to determine statistical significance. A *P* < 0.05 was considered significant (^*^), <0.01 highly significant (^**^), and <0.001 (^*^^*^^*^). For multiple comparisons, ANOVA analysis was performed. Results presented with error bars represent mean ± SEM.

## Results

### *Trypanosoma cruzi* and HIV-1 are able to infect astrocytes even simultaneously but carrying an impairment on the HIV infection rate

**Figure 1:**
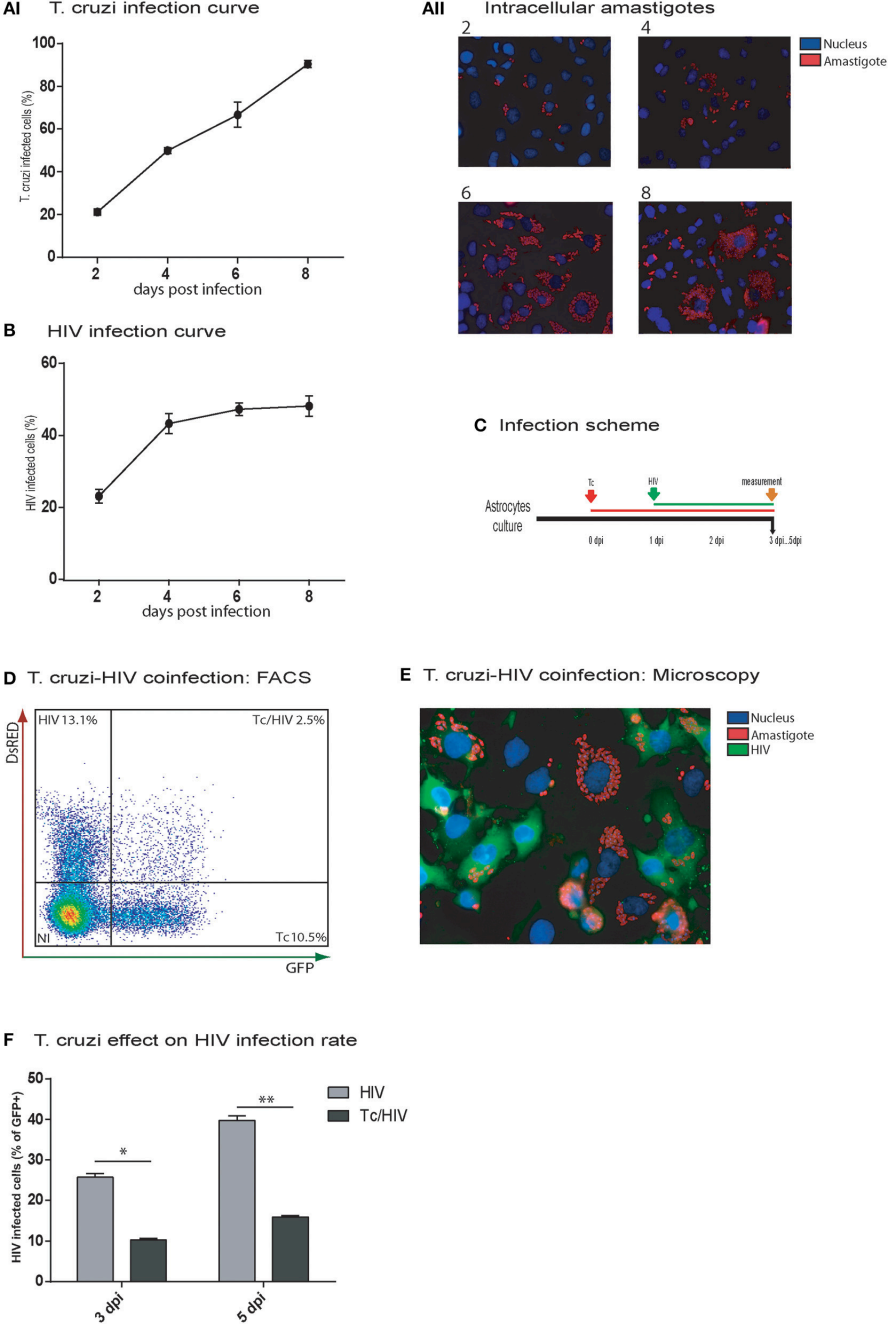
*In vitro* HIV and *Trypanosoma cruzi* infection of astrocytes. Astrocytes were separately exposed to Tc **(AI,II)**, and HIV **(B)**, and their monoinfection curves were determined by intracellular immunostaining and cytofluorimetric analyses at the indicated time points, respectively. The frequency of Tc **(AI)**, the intracellular amastigotes burden **(AII)** and HIV **(B)**-infected astrocytes are represented; figures **(AI,B)** show mean values ± SD of two experiments performed in triplicate. Then, Tc/HIV coinfection assays were performed according to the timeline depicted in **(C). (D)** Flow cytometry was used to characterize Tc and/or HIV infection status of cultured astrocytes by determining the frequencies of Tc-DsRED^+^, and HIV-GFP^+^ cells. **(E)** Fluorescence microscopy showing Tc (Red: K98- Alexa fluor 647-labeled intracellular amastigotes) and/or HIV (Green: p24 FITC-labeled) infected-astrocytes at 5 dpi. Cell and parasite nucleus were stained by DAPI (blue). **(F)** The impact of Tc coinfection on the HIV-infection rate (measured by cytofluorimetric analysis as percentages of GFP^+^ cells) at 3 and 5 dpi is represented. Significant differences were assessed by an unpaired *t*-test and are indicated by ^*^P < 0.05 and ^**^P < 0.01, respectively.

HIV and *T. cruzi* are able to infect separately human astrocytes (Churchill et al., 2009; Vargas-Zambrano et al., 2013). Our first aim was to elucidate their capability to coinfect human astrocytes, even their cellular coexistence, as well as the reciprocal impact on their infection rate. For these goals, we examined the infection rate in three distinguishable conditions, including each pathogen monoinfection, and coinfection. First, the monoinfection rate of *T. cruzi* in astrocytes (evaluated as percentage of cells exhibiting amastigotes) and the number of amastigotes per infected cell were measured at 2, 4, 6, and 8 dpi. The first amastigotes were observed at 2 days after exposure to *T. cruzi* (strain CL, or K98). Figure 1AI shows the percentage of astrocytes infected by *T. cruzi*, whereas Figure 1AII illustrates the intracellular amastigotes increase over time, and incipient trypomastigotes release at 8 dpi.

Regarding the HIV monoinfection, the infection rate on astrocytes at 2 dpi reached 20.0 ± 1.5% (measured as intracellular p24 antigen expression) and was increased up to 43.3 ± 2.7% at 4 dpi; such a rise continued slightly (48.2 ± 3.1) at 8 dpi (Figure 1B).

In double infection assays, when astrocytes were first exposed to *T. cruzi* and then to HIV, (Figure 1C), at 5 dpi, *T. cruzi* and HIV cellular cohabitation was clearly evidenced by both flow cytometry (Figure 1D) and fluorescence microscopy (Figure 1E). However, this phenomenon was observed with a significantly lower frequency (2.5 ± 0.5%) in comparison with HIV (13.1 ± 1.7%) and *T. cruzi* (Tc 10.5 ± 1.7%) monoinfections (Figure 1D). Furthermore, a significant diminishing was denoted in the HIV infection rate at 3 dpi (HIV 25.7 ± 1.0% vs. Tc/HIV 10.2 ± 0.5%) and 5 dpi (HIV 37.7 ± 1.2% vs. Tc/HIV 15.9 ± 0.4%; Figure 1F).

### Cytoprotection is observed on astrocytes infected with *Trypanosoma cruzi* and exposed to HIV

**Figure 2:**
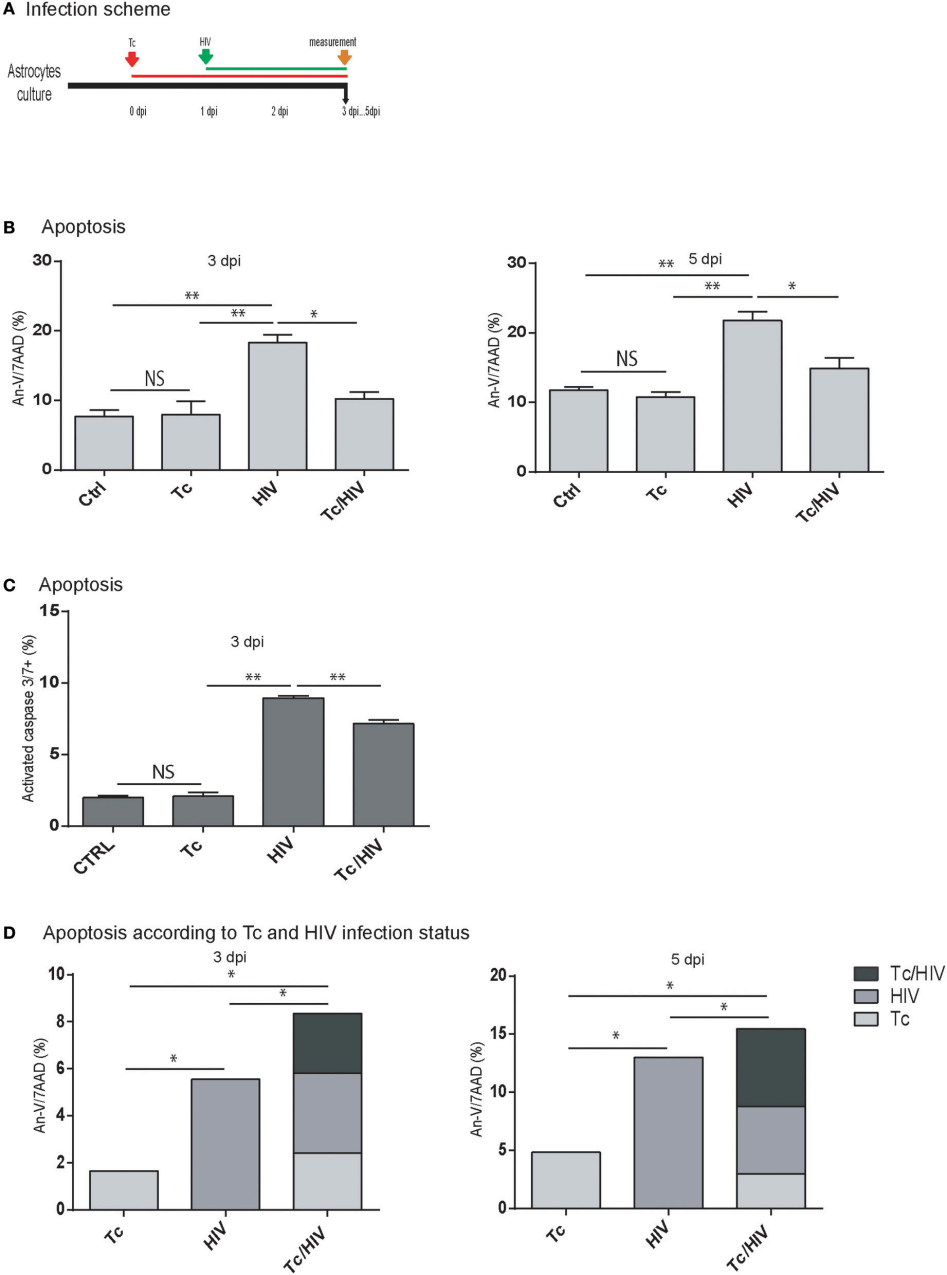
Apoptosis of astrocytes after *Trypanosoma cruzi* and HIV exposure. **(A)** Infection timeline **(B)** Flow cytometric apoptosis assay (Annexin V/7AAD) on astrocytes upon exposure to Tc, HIV, or combination (Tc/HIV). Percentage of apoptotic cells at 3 and 5 dpi is shown. Mean values ± SD from three different experiments. Statistical significance for each condition vs. pathogen unexposed control, or between conditions is indicated by asterisk(s). Two-tailed paired Student *t-*test *P*-values indicate statistical significance (^*^P < 0.05 and ^**^P < 0.01). **(C)** Caspase 3–7 activation measured by FLICA (fluorescently labeled inhibitors of caspasas) combined with plasma membrane permeability assessment (propidium iodide; PI) after exposure to Tc, HIV, or both (Tc/HIV) (^**^P < 0.01 and ^*^P < 0.05 compared with the pathogen unexposed control). **(D)** Flow cytometric apoptosis assay (Annexin V-APC/7AAD) on astrocytes distinguishing for each productively-infected pathogen status (Tc, HIV, and Tc/HIV) at 3 and 5 dpi.

At this point, it was relevant to accomplish whether the diminished rate of HIV infection on astrocytes was related to the increased cellular apoptosis during the coinfection. For this aim, the level of apoptosis was evaluated among astrocytes in the three experimental conditions (HIV monoinfection, *T. cruzi* monoinfection, and *T. cruzi*/HIV coinfection following the scheme of Figure 2A). For this purpose, Annexin-V/7AAD and the active forms of caspase-3 and -7 were measured. In HIV monoinfection, Annexin-V/7AAD level at 3 and 5 dpi (17.8 ± 1.6 and 20.7 ± 0.1%, respectively) was significantly higher than *T. cruzi*/HIV coinfection (9.9 ± 1.0 and 14.9 ± 1.5%, respectively) and *T. cruzi* monoinfection (7.9 ± 1.9 and 11.0 ± 0.8%, respectively; Figure 2B). Similarly, the caspase-3 and -7 activation level was significantly higher in HIV monoinfection (8.9 ± 0.2%) than *T. cruzi*/HIV (7.2 ± 0.3%) and *T. cruzi* monoinfection (2.1 ± 0.3%) at 3 dpi (Figure 2C). The apoptosis level among *T. cruzi* monoinfected astrocytes exhibited no differences in comparison with non-exposed cells.

During HIV and/or *T. cruzi* astrocytes infection, cells are accompanied by non-infected neighbor ones, as observed in Figures 1D,E. To evaluate and discriminate their apoptosis level, astrocytes were exposed to HIV-DsRed and *T. cruzi*-GFP, and apoptosis was measured with Annexin-V (APC)/7AAD. This strategy allowed us to discern cell death among infected and bystander (exposed, non-infected) cells (Figure 2D). At 3 dpi, the total level of apoptosis among astrocytes exposed to HIV was 12.7 ± 0.1% of which 5.5 ± 0.8% was contributed by the productively HIV-infected cells. Among *T. cruzi* infected cells, the total apoptosis level was 6.8 ± 0.7%, of which 1.6 ± 0.2 was due to infected astrocytes. In *T. cruzi*/HIV assays, total apoptosis level was 10.5 ± 0.9%, where three distinguishable scenarios are observed: productively coinfected-cells contributed with 2.5 ± 0.4%, mono infected *T. cruzi* astrocytes with 2.4 ± 0.1%, and monoinfected HIV cells with 3.4 ± 0.4%. Therefore, non-infected astrocytes suffer apoptosis as a consequence of a bystander effect induced by pathogens exposition: HIV: 7.7 ± 1.1%, Tc: 4.5 ± 0.2%, and Tc/HIV: 3.6 ± 0.8%.

Similarly, at 5 dpi (Figure 2D) astrocytes exposed only to HIV depicted a total apoptosis level of 21.9 ± 0.2% (13.0 ± 0.8% among productively infected cells); cells exposed only to *T. cruzi* exhibited a total apoptosis level of 10.4 ± 0.2% (including a 4.8 ± 0.9% among parasite-infected cells). The total apoptosis level of astrocytes exposed to both pathogens was 19.5 ± 0.4% that included those found in HIV, *T. cruzi*, and *T. cruzi*/HIV productively infected-cells (5.8 ± 0.1, 3.0 ± 0.3, 6.6 ± 0.3%, respectively). The contribution to apoptosis due to the bystander effect induced by HIV, *T. cruzi*, or both was 9.2 ± 0.5, 6.3 ± 0.2, and 4.9 ± 0.4%, respectively.

### HIV-*Trypanosoma cruzi* coinfection induces IL-6 overexpression which influences apoptosis level

**Figure 3:**
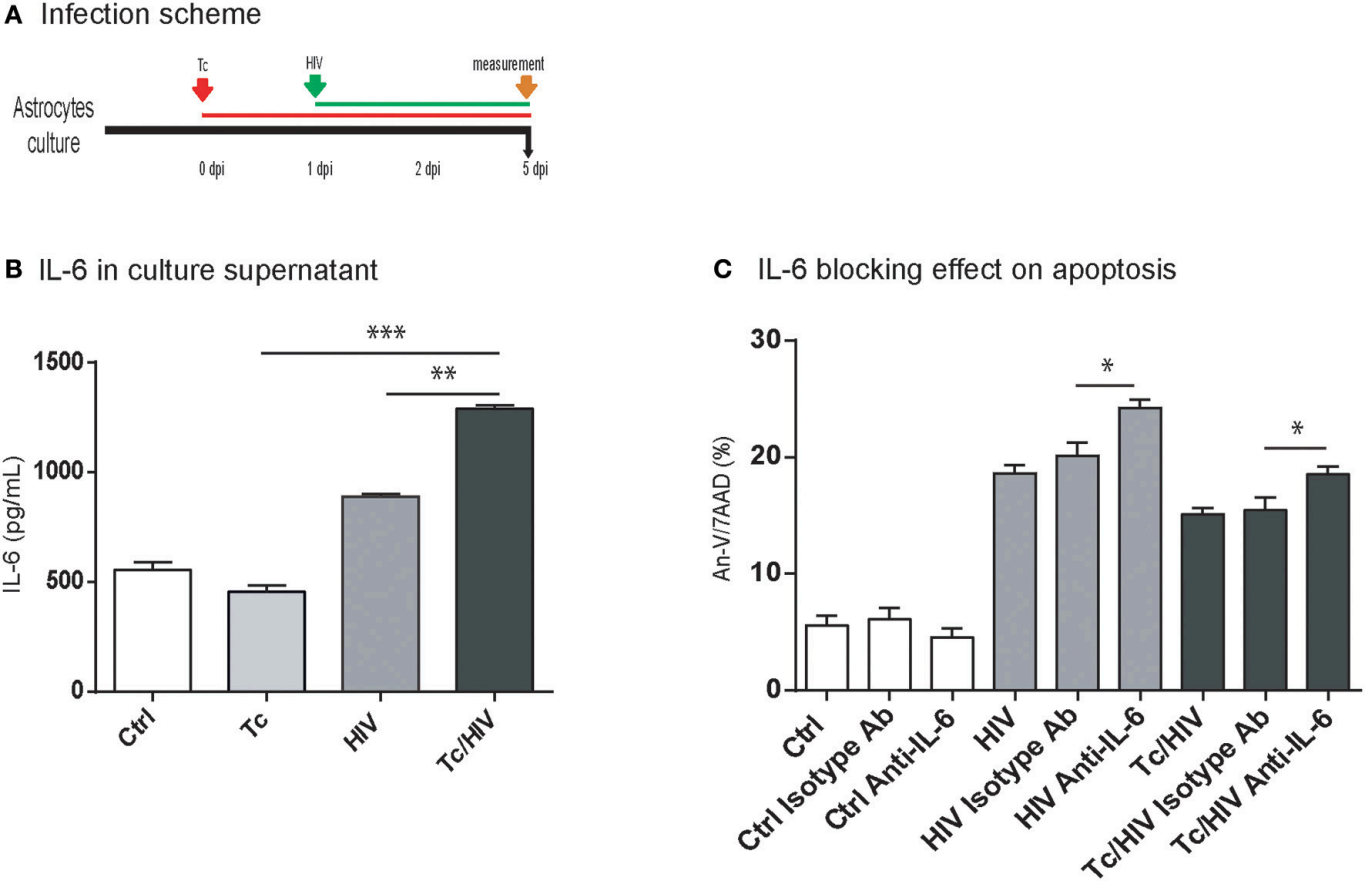
IL-6 level according to infection status and effect of IL-6 blocking assay on apoptosis. **(A)** Infection timeline. **(B)** IL-6 levels were evaluated in culture supernatant by ELISA at 5 dpi for each condition. **(C)** Flow cytometric apoptosis assay (Annexin V/7AAD) on astrocytes upon exposure to Tc, HIV, or combination (Tc/HIV). For each condition is also shown the IL-6 blocking assay. IgG1 isotype controls (“isotype Ab”) were included for each condition. Percentage of apoptotic cells at 5 dpi is shown. Mean values ± SD from three different experiments. Statistical significance for each condition vs. pathogen unexposed control, or between conditions is indicated by asterisk(s). Two-tailed paired Student *t*-test *P*-values indicate statistical significance (^*^P < 0.05, ^**^P < 0.01, and ^***^P < 0.001).

The HIV replication is strongly related to the level of cellular activation. Such a cellular status is, among others, associated with the type and level of circulating cytokines (Rosenberg et al., 1997). The concentration of IL-1β, IL-2, IL-10, IL-4, IL-6, IFN-γ, TNF-α, and IL-17A cytokines was quantified at 5 dpi in astrocyte culture supernatants in the three experimental conditions (each monoinfection, and coinfection; Figure 3A). IL-6 was the only one to reach quantifiable levels for both HIV monoinfection and *T. cruzi*/HIV coinfection scenarios. Significant higher levels in coinfected astrocytes were found (1293.4 ± 13.8 pg/mL) compared to those exposed only to HIV (860.0 ± 5.0 pg/mL), or *T. cruzi* (424.2 ± 57.3 pg/mL; Figure 3B).

Among the different conditions assayed, significantly higher levels of apoptosis (Annexin-V/7AAD) were observed after blocking IL-6 using a neutralizing monoclonal antibody:(i) HIV-monoinfected cells (24.2 ± 0.7 vs. 18.6 ± 0.7%), and (ii) HIV/Tc coinfected astrocytes (18.6 ± 0.6 vs. 15.1 ± 0.6%; Figure 3C). Thus, considering that IL-6 participates on cytoprotection, its overexpression may contribute to the reduction of apoptosis observed during *T. cruzi*/HIV coinfection.

### *Trypanosoma cruzi* infection alters the HIV replication in astrocytes

**Figure 4:**
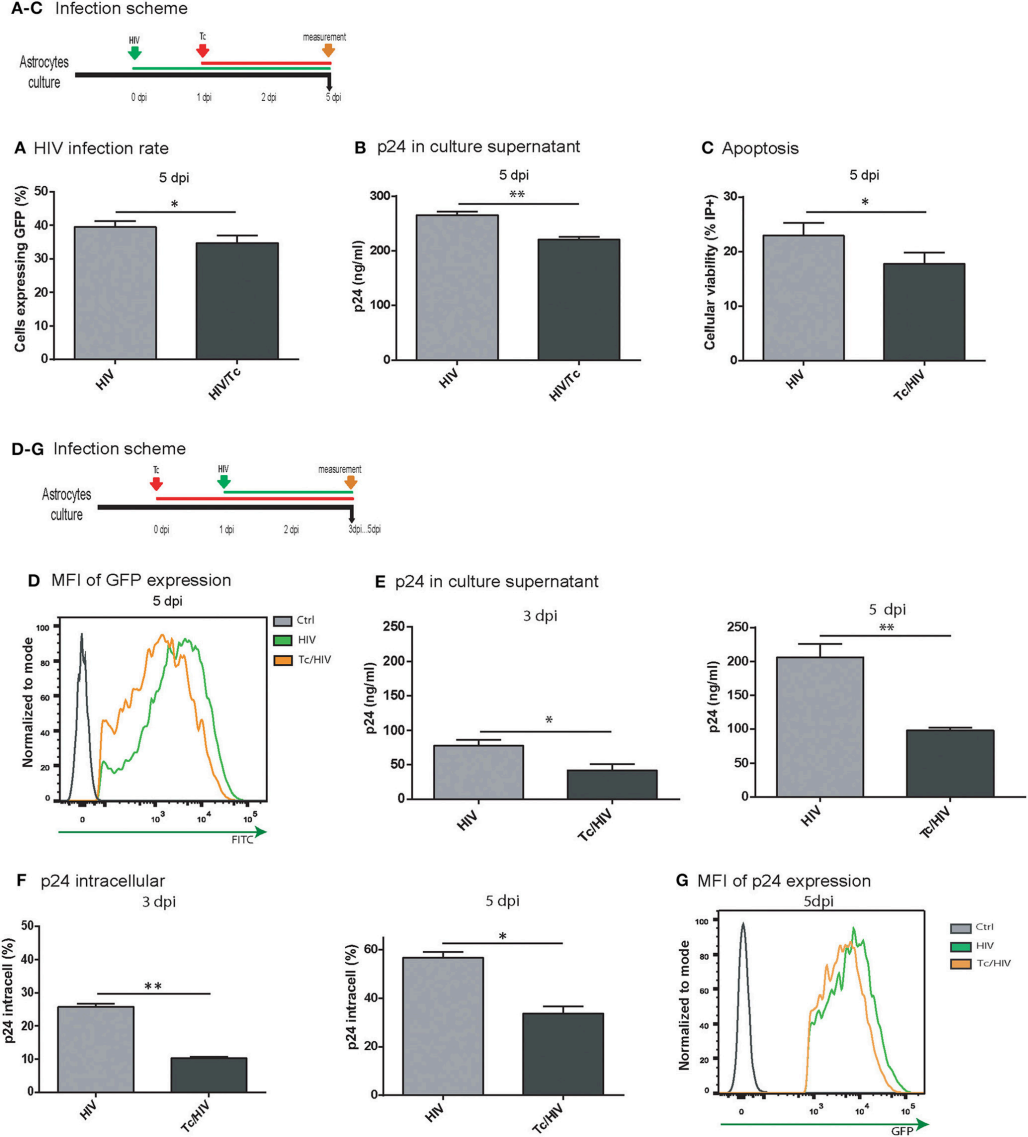
*Trypanosoma cruzi* impairs HIV-1 replication in astrocytes. Effect of Tc superinfection on HIV replication rate measured by cytofluorimetric analysis as GFP^+^ cells **(A)**, and by p24 level in culture supernatant expressed in ng/ml) measured by ELISA **(B)** at 5 dpi. **(C)** Cell viability measured by flow cytometry as the percentage of propidium iodide positive cells (PI^+^) at 5 dpi. (^**^P < 0.01 and ^*^P < 0.05). **(D)** Quantitative analysis of HIV-GFP positive cells proportion and mean fluorescence intensity (MFI) measured by flow cytometry at 5 dpi. **(E)** Tc impact on HIV replication rate measured as p24 level (in culture supernatant expressed in ng/ml) at 3 and 5 dpi. **(F)** Tc impact on HIV replication rate measured by flow cytometry analysis of intracellular p24-FITC expression at 3 and 5 dpi (^**^P < 0.01 and ^*^P < 0.05). **(G)** Quantitative analysis of p24-FITC^+^ cells and mean fluorescence intensity (MFI) measured by flow cytometry at 5 dpi.

As we described above, HIV infection rate is altered in an established *T. cruzi* infection. In order to elucidate if parasite modify astrocytes permissiveness to viral infection, two experimental approaches were carried out. First, we inverted the order of the timeline of pathogen exposure (Figure 4). Hence, astrocytes were first exposed to HIV, and 24 h later to *T. cruzi;* measurements were carried out at 5 dpi. Under this schedule, both HIV infection rate (measured as intracellular GFP-expression) and its level of replication (measured as p24 production in culture supernatants) were significantly lower when *T. cruzi* coexists (HIV 39.5 ± 1.8% vs. HIV/Tc 34.8 ± 2.2% and HIV 280.7 ± 6.5 ng/ml vs. HIV/Tc 220.4 ± 5.4 ng/ml, respectively; Figures 4A,B). Such detrimental effects appear to be parasite-dependent considering that cell viability was preserved -or even it was higher- among coinfected astrocytes in comparison with HIV-monoinfected cells (Figure 4C). These observations prompted us to conclude that the presence of *T. cruzi* affects HIV replication irrespectively of their order to infect astrocytes.

Second, it is expected that according to *T. cruzi* infection progress (increment of infected cells and number of amastigotes per cell) its detrimental influence on HIV replication should be more pronounced. For this goal, we compared the level of viral replication between HIV-monoinfected and HIV-*T. cruzi* coinfected astrocytes at 3 and 5 dpi, following the scheme Figures 4D–G. This procedure was performed using (a) HIV-expressing GFP with further flow cytometry evaluation, and (b) quantification of the HIV p24 antigen expression at both intracellular (by flow cytometry) and extracellular (by ELISA) levels. As described above (Figure 1F), the flow cytometry analysis revealed that the percentage of HIV-infected cells was significantly lower in the *T. cruzi*/HIV condition at 3 dpi, and even more at 5 dpi. At the individual cell level, the quantitative measurement of HIV replication was inferred by the mean fluorescence intensity (MFI). A significant reduction was also observed when HIV and *T. cruzi* coexist (HIV 3588.0 ± 263.0 vs. Tc/HIV 1417.5 ± 30.0, at 5 dpi; Figure 4D). Similarly, the expression level of extracellular p24 antigen was lower in HIV/*T. cruzi* (98.0 ± 4.0 ng/ml) than in HIV-monoinfection (206.0 ± 19.0 ng/ml; Figure 4E, at 5 dpi). Coincidentally, the rate of cells expressing intracellular p24 antigen (HIV 25.0 ± 0.7% vs. Tc/HIV 11.5 ± 0.5%; Figure 4F, at 3 dpi) as well as the quantitative expression level of intracellular p24 (as MFI: HIV 7638.5 ± 183.1 vs. Tc/HIV 5094.0 ± 43.8; Figure 4G) were also markedly diminished in *T. cruzi* co-presence.

### HIV replication modulated by *Trypanosoma cruzi*: effect of benznidazole treatment and parasite multiplication rate

**Figure 5:**
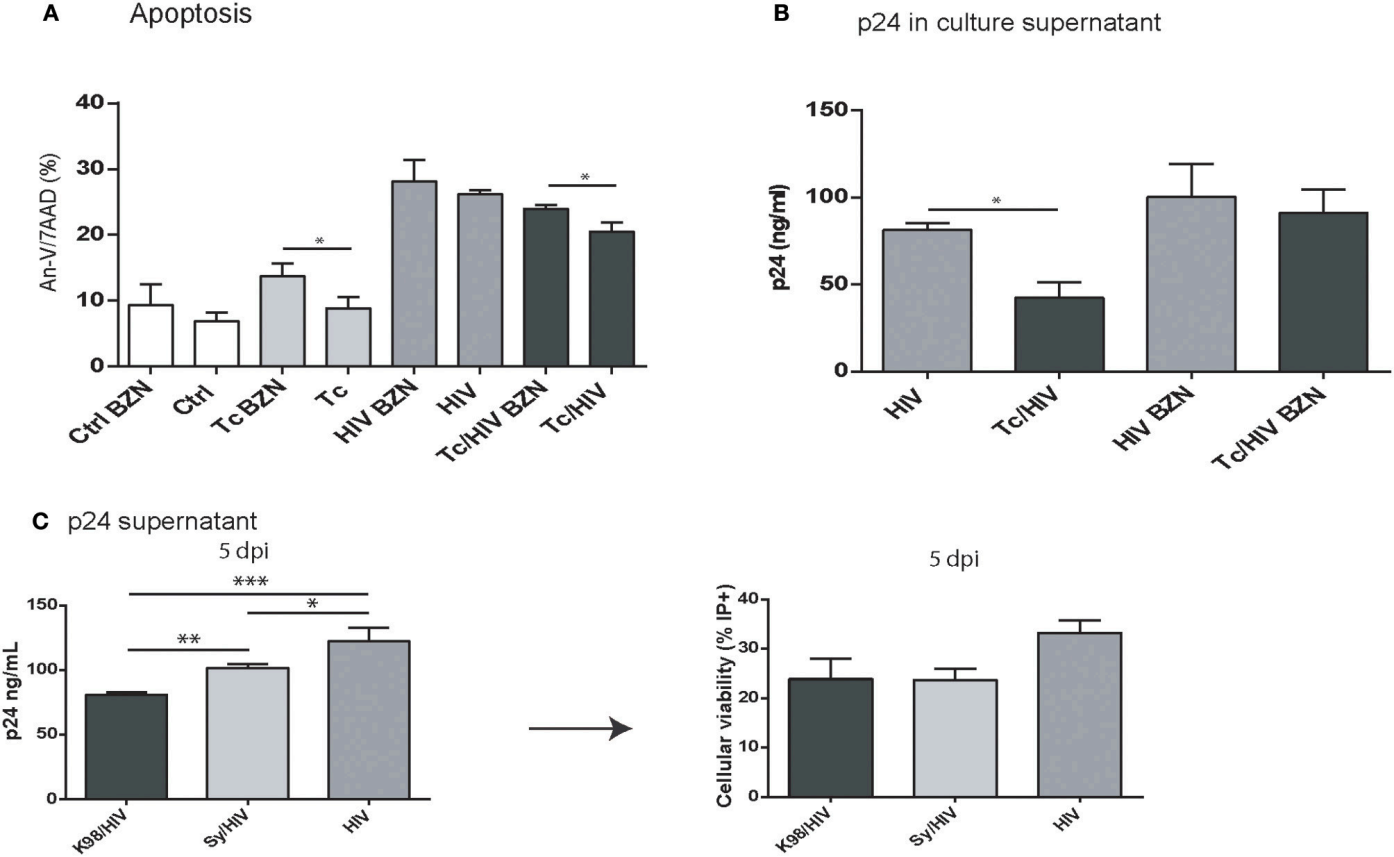
**(A,B)** Benznidazole (BZN) effect on apoptosis and viral replication in astrocytes exposed to *T. cruzi* and HIV. **(A)** Flow cytometric apoptosis assay (Annexin V/7AAD) on astrocytes upon exposure to Tc, HIV, or combination (Tc/HIV) treated (or not) with BZN. Percentage of apoptotic cells at 3 dpi is shown. **(B)** Comparative HIV replication measured as p24 level concentration in culture supernatant (by ELISA) in monoinfected, and *T. cruzi*-coinfected astrocytes treated (or not) with BZN. **(C)** Comparative analysis of different Tc parasites (K98, Sy) impact on HIV replication and cell viability (measured by ELISA as p24 culture supernatant, and propidium iodide positive cell -PI^+^- levels, respectively). Mean values ± SD from three different experiments. Significant differences were assessed by an unpaired *t*-test and are indicated by (^*^P < 0.05, ^**^P < 0.01, and ^***^P < 0.001).

The parasite-related downregulation on the HIV replication on astrocytes may be related to *T. cruzi* multiplication rate. To analyze this association we inhibited *T. cruzi* multiplication using benznidazole (BZN), a trypanocidal agent. Then, both apoptosis level (Annexin-V/7AAD) and HIV replication (p24 antigen in supernatant) were measured among astrocytes in the three experimental conditions (HIV monoinfection, *T. cruzi* monoinfection, *T. cruzi*/HIV coinfection). After BZN treatment the level of cell death was increased when comparing against BZN-untreated *T. cruzi*-infected astrocytes (Tc monoinfection: 13.7 ± 2.0 vs. 8.8 ± 1.8%; Tc/HIV coinfection: 24.3 ± 0.5 vs. 20.5 ± 1.4%, with and without BNZ, respectively; Figure 5A). In line with this, HIV replication level was significantly higher in BZN-treated HIV/Tc coinfected cells than untreated cells (91.0 ± 9.5% vs. 42.2 ± 12.8 ng/ml). The HIV replication among HIV-monoinfected cells exhibited no differences regarding the BZN treatment (Figure 5B).

Furthermore, other approach was carried out to study the relationship between parasite multiplication and HIV replication. We compared the impact of two different *T. cruzi* strains (K98 and Sylvio), that display different multiplication rate in astrocytes (measured as number of amastigote per cell). Fixing the astrocyte:Sy (Sylvio) inoculum to 1:10, and K98 to 1:5 similar infection rates were achieved (22.1 ± 3.7%) but multiplication rate was significantly lower for Sy than K98 parsaites (7.2 ± 3.8 and 21.1 ± 7.4 amastigote/cell, respectively). Then, astrocytes were exposed to equal HIV inoculum and the viral replication was measured (level of extracellular p24 in culture supernatants). The HIV replication in monoinfection was 122.3 ± 10.8 ng/mL, whereas in coinfection assays significant decreases were observed. While Sy strain induces viral replication diminishing (101.7 ± 2 ng/mL), K98, a more replicative strain, induces a more pronounced effect (80.9 ± 1.9 ng/mL; Figure 5C). Altogether, our results strongly suggest that *T. cruzi* impairment on HIV replication is associated to parasite multiplication.

### *Trypanosoma cruzi* does not alter the infectivity of the HIV progeny

**Figure 6:**
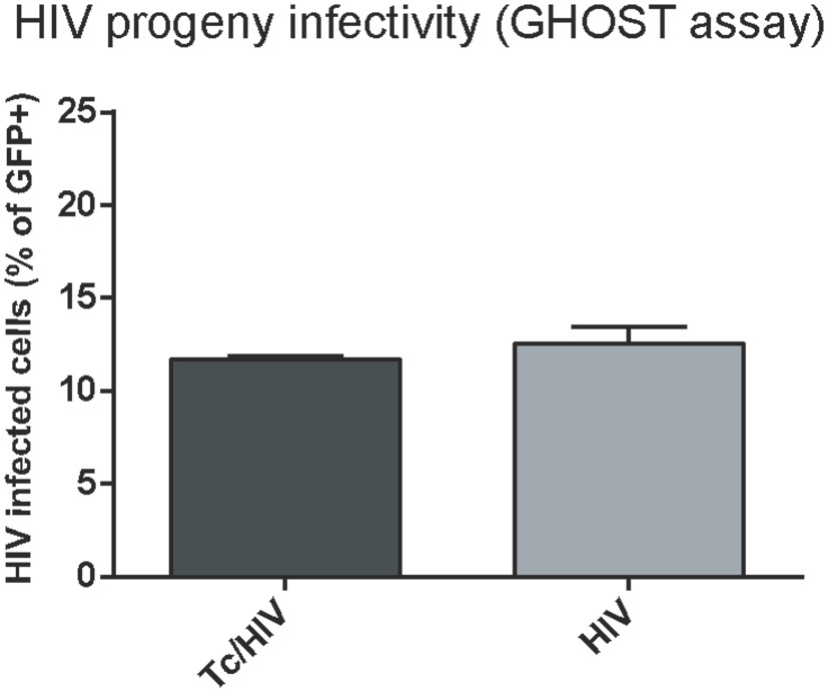
*Trypanosoma cruzi* effects on infectivity of the HIV progeny. GFP expression in GHOST cell culture inoculated with supernatant from HIV and Tc/HIV was measured by flow cytometry at 3 dpi. Mean values ± SD from three different experiments. No significant differences were found between the two conditions (>0.05).

The reduced HIV replication could also be ascribed to diminished infectivity when some defects in the viral progeny are caused. We further examined the plausible *T. cruzi* influence on HIV infectivity using GHOST cells and flow cytometry. No significant differences in the infectivity of the HIV progeny were found at 3 days post-exposition, thus inferring that *T. cruzi* does not modify the replication efficiency of the HIV progeny (Figure 6).

### Soluble factors are involved in HIV replication impairment by *Trypanosoma cruzi*

**Figure 7:**
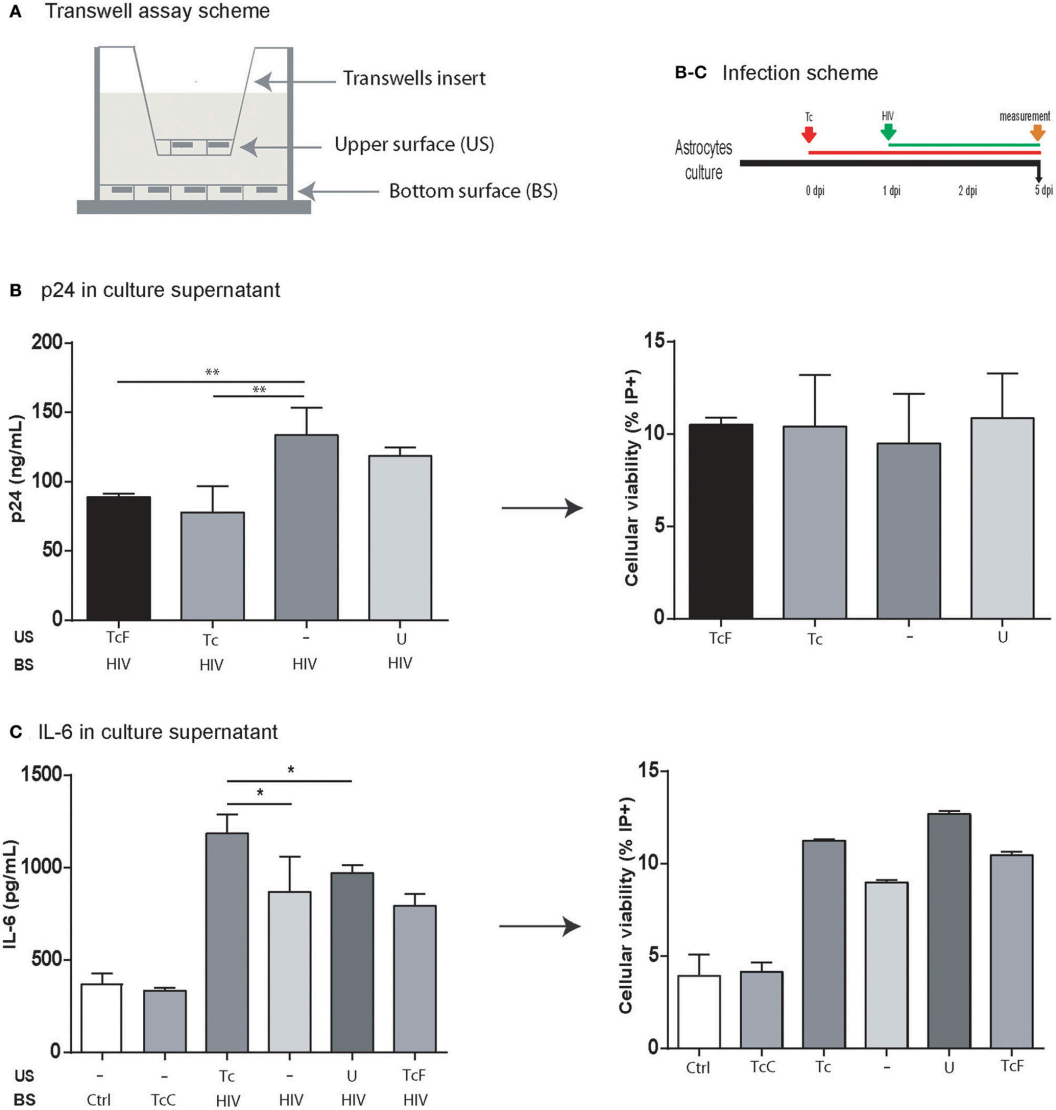
Impact of the soluble factors on HIV replication and IL-6 level. **(A)** Schematic depiction of the setup for a transwell assay with the upper surface (US) separated from the bottom surface (BS) by a membrane with 0.4-μm pores. **(B)** p24 antigen level (ng/mL) in culture supernatant in four different conditions. All cells in the bottom surface were infected with HIV. Four different conditions were fixed to the upper surface: (i) *T. cruzi* free stage (trypomastigote) (TcF), (ii) astrocytes infected with *T. cruzi* (Tc), (iii) empty (−), and (iv) uninfected astrocytes (U). The cell viability in the bottom surface was measured by propidium iodide (PI^+^) by flow cytometry at 5 dpi (^**^P < 0.01). **(C)** IL-6 level in the culture supernatant was measured in six different conditions. In the bottom surface, cells were infected with HIV (+) except for two of them (Ctrl: uninfected cells; TcC: cells infected with Tc only). In the upper surface, astrocytes infected with *T. cruzi* (Tc), empty (−), uninfected astrocytes (U), and *T. cruzi* free stage (trypomastigote) (TcF). The cell viability in the bottom surface was measured by propidium iodide (PI^+^) by flow cytometry at 5 dpi (^*^P < 0.05, and ^**^P < 0.01).

As we described previously, *T. cruzi* decreases the HIV replication. It is hardly associated with HIV-*T. cruzi* cohabitation, due to its low frequency observed. To assess if cell-to-cell contact is needed to attain this effect, we used a transwell® device that define two distinguishable cellular environments (Figure 7A). Astrocytes infected with HIV in the bottom chamber were exposed to the transwell chamber with (i) *T. cruzi*-infected astrocytes, and (ii) free trypomastigotes. As control, uninfected astrocytes were included. At 5 dpi, HIV replication (as extracellular p24 level) and apoptosis were measured in the bottom chamber. The HIV replication (133.6 ± 19.9 ng/mL) was significantly lower when they were exposed to *T. cruzi*, either parasite-infected astrocytes (77.9 ± 18.7 ng/mL), and free-parasites (92.8 ± 9.0 ng/mL). Hence, both *T. cruzi* stages (intracellular amastigotes and extracellular trypomastigotes) are able to alter viral replication remoteness (Figure 7B). Considering IL-6 overexpression findings (Figure 3) we evaluate it following the same transwell device. The IL-6 level from HIV-monoinfected astrocytes (867.9 ± 190.3 pg/mL) were increased when HIV-infected astrocytes were opposed to *T. cruzi* infected cells (1186.2 ± 101.4 pg/mL) but not when were opposed to free-trypomastigotes (334.7 ± 12.9 pg/mL; Figure 7C).

Taken together, IL-6 upregulation is independent of HIV-*T. cruzi* intracellular cohabitation and cell-to-cell contact. On the other hand, the influence of *T. cruzi* on HIV replication depends on soluble factors released by parasites either from infected cells and/or by trypomastigotes.

### *Trypanosoma cruzi* infection reduces herpes *Simplex* type-2 (HSV-2) replication

**Figure 8:**
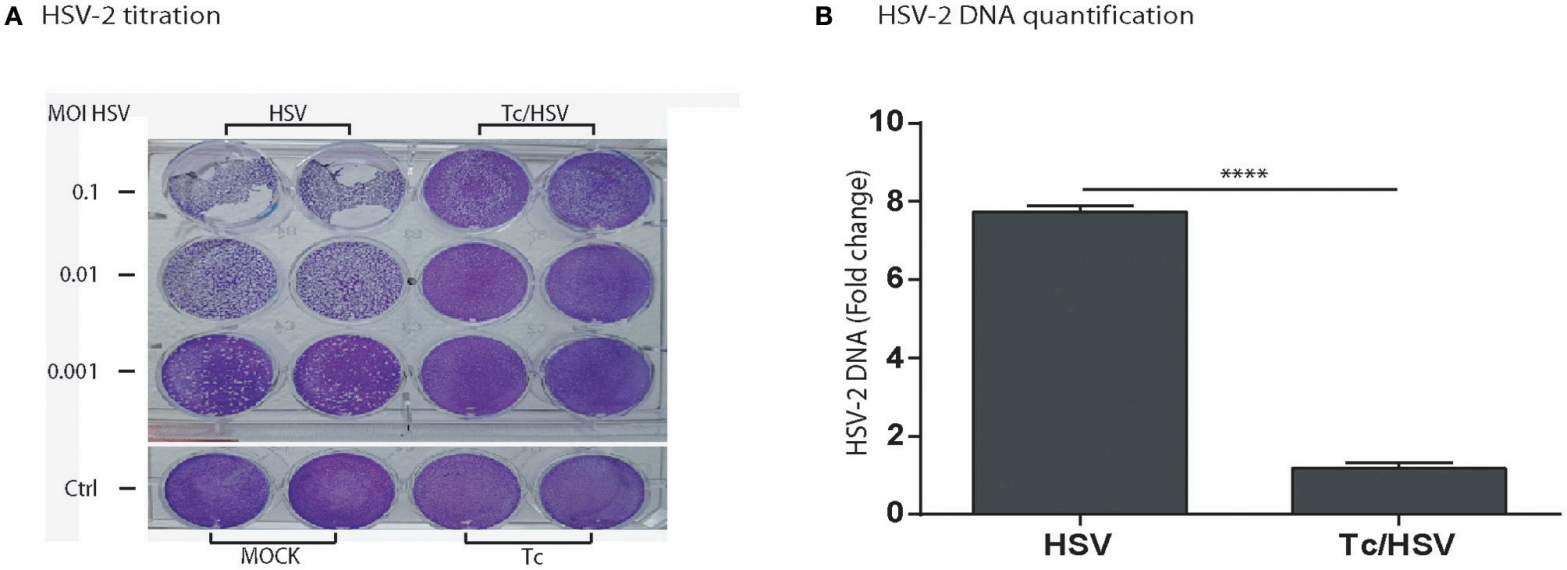
*Trypanosoma cruzi* infection impairs herpes *simplex* type-2 (HSV-2) replication. **(A)** HSV-2 titration by plaque-forming-units assay in Vero cells showing plaques developed by HSV-2 (G-strain) in Vero cell cultures 48 h after viral monoinfection (columns identified as “HSV”), or parasite + viral coinfection (columns identified as “Tc/HSV”) and stained with crystal violet, as described in Section Materials and Methods. Each line represents an HSV-2 inoculum or multiplicity of infection –m.o.i- (0.1; 0.01; 0.001). “Ctrl”: Non-infected cells (“MOCK”) and *T. cruzi* mono-infected cells (“Tc”). **(B)** Fold-change in HSV-2 DNA between HSV-2 monoinfection, and Tc/HSV-2 coinfection. Two-tailed paired Student *t*-test *P*-values indicate statistical significance (^***^P < 0.001).

In order to elucidate that *T. cruzi* infection diminishes viral replication not only for HIV, we assessed the parasite effect on viral replication for HSV-2, as alternative neurotrophic virus. Viral replication was measured using a semiquantitative viral plaque assay and quantification of viral DNA in cell culture supernatant. The magnitude of cytophatic effect on Vero cells (cellular lysis) decreased proportionally according to the three HSV-2 inoculums (m.o.i) being maximum [++++] for m.o.i = 0.1, intermediate [+++] for m.o.i = 0.01, and low [++] for m.o.i = 0.001. The cellular lysis grade decreased substantially for each viral inoculum (from very low [+] to not visible [0], respectively) when Vero cells were previously exposure to *T. cruzi*. Similarly, a marked lower (7.6 fold-change) HSV-2 DNA was detected when *T. cruzi* and HSV-2 coinfected Vero cells in comparison with HSV-2 monoinfection (Figure 8).

## Discussion

The natural history of *T. cruzi* infection could be modified by immunosuppression in most patients with Chagas disease predisposing them to a reactivation episode. In them, a more severe clinical presentation (e.g., central nervous system involvement and severe myocarditis) is observed compared to immunocompetent patients with acute Chagas disease (Pinazo et al., 2013). Mass lesions in the white matter of the brain are the most frequent clinical findings, but meningitis and meningoencephalitis may also occur (Sica et al., 2008; Pinazo et al., 2013). This CNS involvement was reported in 75–80% of patients coinfected with *T. cruzi* and HIV, being more frequent than myocardial damage (Sartori et al., 1995, 2007; Cordova et al., 2008). Reciprocal interactions between both pathogens are suspected to occur in CNS cells but to date no data is available yet.

Glial cells are an important source of CNS cytokines and chemokines and they might likely contribute actively in the physiopathology of *T. cruzi* infection. In murine models, glia but not neurons harbor amastigotes (Roffe et al., 2003; Rachid et al., 2010). On the other hand, the astrocytoma is the only CNS human cell line reported to be susceptible to *T. cruzi* infection (Vargas-Zambrano et al., 2013). Astrocytes, the main cells in brain tissues (Seth and Koul, 2008), participate in the environment maintenance of neurons as well as in immune functions producing cytokines and chemokines. They can host several infectious agents, including HIV, appearing to be the most abundant HIV-infected cell of the CNS (Dewhurst et al., 1987; Churchill et al., 2009), therefore contributing predominantly to the neurotoxicity commonly observed during the chronic stage of HIV infection. However, it is unknown whether the coexistence of both pathogens in the same astrocyte is possible, as well as its consequences.

The present study shows that HIV and *T. cruzi* are able to coexist in human astrocytes as a phenomenon that lasts over time. Moreover, such coexistence was possible regardless of the order in which both pathogens entered. However, cellular cohabitation showed a significantly lower frequency than the unique infections of each pathogen. In this scenario, reciprocal interactions between both pathogens could be enhanced considering that their multiplication appeared to occur. In this regard, a *T. cruzi*-mediated detrimental effect on HIV replication was reported previously on macrophages and human placenta cells (Dolcini et al., 2008; Andreani et al., 2009).

It is known that HIV exhibits a pro-apoptotic potential not only among productively infected cells but also among non-infected ones as a bystander effect (Eugenin and Berman, 2013). In contrast, *T. cruzi* displays a cytoprotective role by inhibiting apoptosis in several cell types (Nakajima-Shimada et al., 2000; Schaumburg et al., 2006). Here, we observed that this cytoprotection ability is still conserved under concomitant HIV infection. Interestingly, the cytoprotection was reversed when the parasite development was inhibited by the treatment of *T. cruzi*-infected cells with benznidazole. Furthermore, we observed a significantly diminished HIV progeny size when *T. cruzi* infection occurred as a previous (Figure 4E) or a subsequent event (Figure 4B), as well as *T. cruzi* (Figure 8B) is able to impact negatively on HIV replication irrespectively of their cellular cohabitation and/or infected cell-to-cell contact, or even HIV-infected cell-to-free trypomastigotes contact.

Resident astrocytes are involved in CNS inflammation after trauma or infection (Cooley et al., 2014) by secreting host-cellular factors such as IL-6 (Norenberg, 1994; Van Wagoner et al., 1999). This cytokine is considered a neuropoietin that participates in neurogenesis as well as responding to normal and damage conditions of mature neurons and glial cells (Erta et al., 2012; Liu and Kumar, 2015). We observed that *T. cruzi* monoinfected astrocytes did produce IL-6 similarly to uninfected control cells, in agreement with recently reported results (Duran-Rehbein et al., 2017). However, increased levels of IL-6 were observed in HIV/*T. cruzi* coinfected astrocytes. Such IL-6 level was observed independently of cell coexistence of the pathogens, and cell-to-cell contact as well, thus depending of parasites soluble factors. In agreement, by IL-6 blocking we could induce an increment of apoptosis revealing its participation in the cytoprotection observed.

Both astrocytes infected by neurotropic viruses such as herpes *simplex* (Detje et al., 2015; Lindqvist et al., 2016) or the intracellular amastigotes of *T. cruzi* (Costa et al., 2006; Koga et al., 2006) induce the release type I-IFNs capable of inhibiting HIV-1 replication in cell culture (Ikeda et al., 2015a,b). Here, when we assessed herpes *simplex* virus-*T. cruzi* coinfection in non-producing type I-IFNs Vero cells (Osada et al., 2014) the parasite-related cytoprotective role was also observed. Free trypomastigotes are equally capable of stimulating IFNs-I released via activation of TLR2 through parasite-derived ligands (Campos et al., 2001) that may have an impact on the infection outcome by conditioning the host environment and the immune response. Among these ligands, we evaluated the role of *trans*-sialidase (TS), a trypomastigote well characterized virulence factor secreted in vesicles and involved in immune evasion (Mucci et al., 2017), pathogenesis (Leguizamon et al., 1999; Mucci et al., 2002; Risso et al., 2007; Freire-De-Lima et al., 2015), and also proposed as a neuroprotective factor (Chuenkova and Pereiraperrin, 2011). However, neither inactive nor active TS isoforms (data not shown) appear to propitiate the parasite detrimental effect on HIV replication in astrocytes. Considering that other parasite-secreted proteins may influence the immune response (Watanabe Costa et al., 2016), further research is deserved.

Several studies revealed that different *T. cruzi* Discrete Typing Units (DTUs) participate in human *T. cruzi*—HIV coinfection (Perez-Ramirez et al., 1999; Lages-Silva et al., 2002; Burgos et al., 2005; Hernandez et al., 2014). Moreover, in a multiclonal infection involving DTUs I and V/VI, we reported that CNS histotropism was ascribed to a DTU I population as the etiological agent of meningoencephalitis (Burgos et al., 2008). Here, the *in vitro* detrimental effect on HIV progeny production was shared by two different parasites belonging to DTU I (K98 and Sylvio), but with a dissimilar impact. Hence, under comparable levels of parasite infection rates, the HIV replication decline induced by K98 was higher than the induced by Sylvio strains. This was related to the higher rate of multiplication of K98 strain and, it could be due to the kidnaping of the cellular components during parasite multiplication. Considering that both *T. cruzi* and HIV replicate intracellularly, they depend on the host cell energy at adequately high rates. These pathogens hijack cells in order to multiply, and efficient multiplication needs a metabolite-enriched host. Therefore, *T. cruzi* could diminish apoptosis of HIV-infected cells which may impact on the establishment of reservoirs. Comparatively, whether *T. cruzi* exhibits higher consumption of energy and metabolites than HIV is not known, but have observed that when parasite multiplication diminishes it allows higher HIV replication. Furthermore, the same parasite-mediated downregulation on the HIV replication was observed with two different *T. cruzi* parasite populations (K98 of DTU I and CL of DTU VI) genetically distant but sharing similar replication rates in the system used (data not shown). These data were further supported when we treated HIV-*T. cruzi* coinfected astrocytes with benznidazole, leading to viral replication recovery. These observations suggest that the *T. cruzi* multiplication rate could be a relevant factor in driving HIV progeny.

In conclusion, human astrocytes can concurrently host both *T. cruzi* and HIV. This scenario favors interactions between both pathogens without the requirement of cohabiting a cell or infected cell-to-cell contact. Moreover, *T. cruzi* decreases both the level of HIV replication as well as astrocyte apoptosis by cellular (IL-6) and parasite-release soluble factors are involved. These data provide novel insights into pathogenesis of *T. cruzi*-HIV coinfection at the CNS level, raising new challenges for future research on parasite-HIV-host interaction.

## Author contributions

JU, JB, DO, CP, ML, JQ: Design of the work; JU, JB, DO, CP, ML, JQ: Acquisition, analysis, and interpretation of data for the work. JU, JQ: Drafting the work; JB, ML, JQ: Revising the draft critically for important intellectual content. JU, JB, DO, CP, ML, JQ: Final approval of the version to be published. JU, JB, ML, JQ: Supervised the project. All authors are in agreement to be responsible for all features of the work in confirming that questions related to the veracity of any part of the work are appropriately investigated.

### Conflict of interest statement

The authors declare that the research was conducted in the absence of any commercial or financial relationships that could be construed as a potential conflict of interest.
